# Lower hydration status increased diabetic retinopathy among middle-aged adults and older adults: Results from NHANES 2005-2008

**DOI:** 10.3389/fpubh.2022.1023747

**Published:** 2022-10-26

**Authors:** Jiayu Zhang, Ziyang Ren, Qiang Zhang, Rui Zhang, Chunmei Zhang, Jufen Liu

**Affiliations:** ^1^Department of Nutrition, Beijing Luhe Hospital, Capital Medical University, Beijing, China; ^2^Institute of Reproductive and Child Health/National Health Commission Key Laboratory of Reproductive Health, Peking University, Beijing, China; ^3^Department of Epidemiology and Biostatistics, School of Public Health, Peking University, Beijing, China

**Keywords:** hydration, retinopathy, osmolality, dehydration, NHANES

## Abstract

**Background:**

Diabetic retinopathy (DR) is a common complication of diabetic patients. Retinal physiological function is affected by hydration status. We aimed to explore the association between hydration status and DR.

**Methods:**

National Health and Nutrition Examination Survey (NHANES) 2005-2008 was used to perform this cross-sectional study. Serum osmolality was used to assess hydration status for all participants and calculated osmolality was evaluated for only older people. DR and its severity were evaluated and graded into mild non-proliferative retinopathy, moderate/severe non-proliferative retinopathy, and proliferative diabetic retinopathy by the Early Treatment for Diabetic Retinopathy Study protocol and NHANES Digital Grading Protocol. Fully adjusted multivariable logistic regression models were used by SAS OnDemand for Academics.

**Results:**

Among the 5,220 United States adults aged 40 or older, compared with the lowest osmolality group, participants with the highest quartile of serum osmolarity had higher odds of DR (OR: 1.371, 95% CI: 1.001–1.876). For participants with DR, the adjusted OR (95 % CI) of moderate/severe non-proliferative retinopathy and proliferative diabetic retinopathy in the higher serum osmolarity group was 2.119 (1.200–3.741) and 7.001 (3.175–15.438), respectively. Furthermore, in older people, higher calculated osmolarity was significantly associated with increased occurrence of DR (OR: 2.039, 95% CI: 1.305–3.186).

**Conclusions:**

Adults with lower hydration status had higher risk of DR, moderate/severe non-proliferative retinopathy, and proliferative diabetic retinopathy. Dehydration in older adults, classified by calculated osmolality, is associated with a higher rate of DR. There was consistent trend in the results between the two methods.

## Introduction

Diabetic retinopathy (DR) is one of the most common complications of diabetes mellitus (DM) and is the major cause of visual loss in working-age adults (1, 2). Although extensive studies and health promotion programs on DM and its complications have been of great concern, it was reported that 103.12 million adults worldwide had DR in 2020, and this number was expected to be 160.50 million by 2045 (3). Furthermore, the age-standardized global prevalence of blindness due to DR has also increased by 14.9% from 1990 to 2020, placing a significant burden on public health (4).

Appropriate hydration status is the basis for healthy metabolism (5). Dehydration, often classified as isotonic or hypotonic dehydration (equal or more solute loss than water) and hypertonic dehydration (greater water loss than solutes), refers to a decrease in total body water when the fluid lost cannot be entirely replenished by fluid intake (6, 7). Previous evidence has indicated that the risks of urological, gastrointestinal, circulatory, and neurological diseases increase with dehydration (8). People who are dehydrated also have higher mortality and lower quality of life if not treated well (9). In addition, hydration status was reported to affect the retinal physiological function (10). For instance, dehydration can contribute to lower peak systolic velocity of the central retinal artery, as well as decreased retinal nerve fiber layer thickness and macular volume, all of which are risk factors of DR (11–14).

It's widely known that polyuria is a common symptom in people with diabetes. With age, the sensation of thirst blunts, primary urine concentration declines, muscle mass decreases, and total body water and fluid stores decrease, which may increase the risks of dehydration (15–18). Furthermore, age-related pathophysiological changes like the concentration change of Na^+^, K^+^, and Cl^−^ and comorbidities such as hyperglycemia, nephrotic syndrome, and adrenal insufficiency may also result in dehydration (10, 19). Hence, older diabetics are more likely to be influenced by fluid and electrolyte balance disturbances, which may lead to a higher risk of hypertonic dehydration and induce further DR (20, 21). However, limited studies have examined the association of hydration status with DR.

To fill these research gaps, we used data from the National Health and Nutrition Examination Survey (NHANES) 2005-2008 to explore the association between hydration status and DR among middle-aged and older United States adults.

## Materials and methods

### Study population

The NHANES is a cross-sectional survey designed to assess the health and nutritional status of the community dwelling populations in the United States. It started in the early 1960s and has been a continuous program since 1999. Every year, the NHANES collected data from a nearly 5,000-person nationally representative sample. Each participant completed the interview, examination and laboratory tests. The National Center for Health Statistics Website (https://www.cdc.gov/nchs/nhanes/) contains the NHANES data that are available to the general public. The NHANES protocols were approved by the National Center for Health Statistics Research Ethics Review Board. All participants provided written informed consent for this survey.

**Figure 1:**
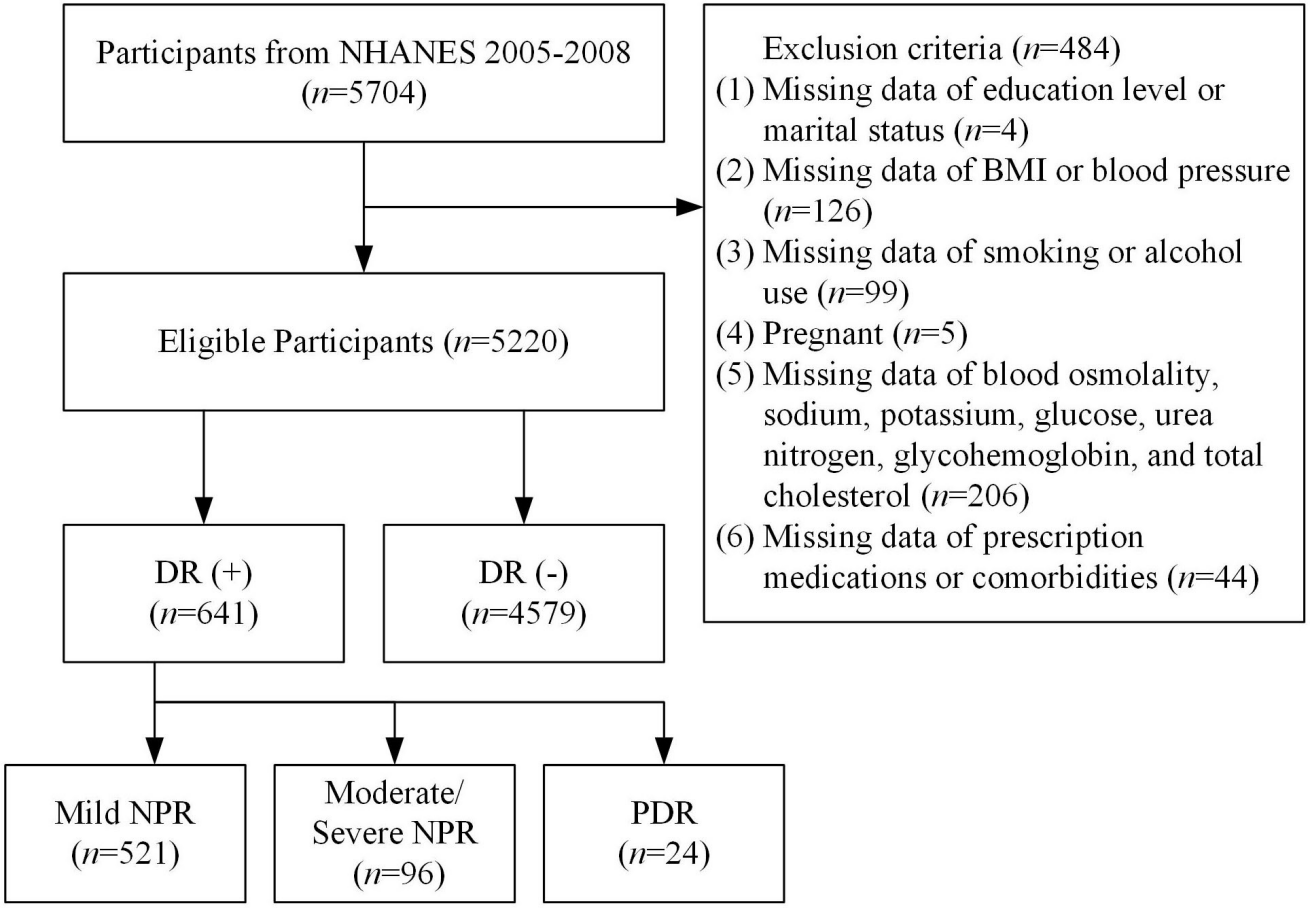
Flow chart of the study.

We used the demographics, dietary, examination, laboratory, and questionnaire data from NHANES 2005–2008. A total of 5,704 participants aged 40 years or older completed the retinal imaging exams. Participants who were blind or had eye infections/patches on both eyes were excluded from this study. According to the flow chart shown in Figure 1, we excluded 4 participants whose education level or marital status questionnaires were incomplete, 126 participants without BMI or blood pressure data, 99 participants without information on smoking or alcohol use, 5 participants who were pregnant, 206 participants without data on blood osmolality, sodium, potassium, glucose, urea nitrogen, glycohemoglobin, and total cholesterol, and 44 participants without information about prescription medications or comorbidities. Finally, 5,220 adults were involved in this study.

### Ascertainment of hydration status

In order to assess and grade the hydration status, serum osmolality was ranked and divided into four groups, the lowest osmolality group (serum osmolality ≤ 275 mOsm/kg), the lower osmolality group (276–279 mOsm/kg), the higher osmolality group (280–282 mOsm/kg), and the highest osmolality group (≥ 283 mOsm/kg) (9, 22).

Considering the impaired pancreatic and renal function of diabetes, we also used calculated osmolarity to evaluate the hydration status of people aged 65 or older for wider application in clinical practice (15, 21–23).


$$\begin{array}{l} Calculated\;Osmolarity=1.86\times \left(Na^{+}+K^{+}\right)+1.15\times glucose\\ \;+urea+14 \end{array}$$

In this equation, blood sodium, potassium, glucose, and urea nitrogen were all measured in mmol/L and were represented by Na^+^, K^+^, glucose, and urea , respectively. Normal hydration (285–294 mmol/L), impending dehydration (295–300 mmol/L), current dehydration (> 300 mmol/L), and hyperhydration (< 285 mmol/L) were the four grades used to categorize the hydration status (23, 24).

### Ascertainment of DR

In participants aged 40 years and older, forty-five-degree non-mydriatic digital retinal images of each eye were captured. EyeQ Lite image processing software was used to view each retinal image. The ophthalmic digital imaging system was used to assess the presence of retinal diseases. DR severity levels were graded by graders at the University of Wisconsin and were classified into no retinopathy, mild non-proliferative retinopathy (NPR), moderate/severe NPR, and proliferative retinopathy (PDR), by the NHANES Digital Grading Protocol (25).

### Covariates

Information on age (40–64 years, ≥ 65 years), sex (male, female), race (non-Hispanic White, other races), education (under high school, high school or equivalent, and above high school), marital status (married: married/lived with partners; not married: widowed/divorced/separated/never married), and smoke status (current, ever, and never) were all obtained by self-reported. Participants who had smoked more than 100 cigarettes in life were considered as current smoking (current smokers) or ever smoking (former smokers); participants who had smoked < 100 cigarettes in life were considered as never smoking. Body mass index (BMI) was calculated as weight (kg) divided by the square of height (m) and was categorized into normal weight (< 25.0 kg/m^2^), overweight (25.0–29.9 kg/m^2^), and obese (≥ 30.0 kg/m^2^), which was measured in the mobile examination centers (MECs). Alcohol consumption (0 gram, 0–20 grams, and >20 grams) was assessed from a 24-h dietary recall. The concentrations of blood glycohemoglobin (< 7.0%, ≥ 7.0%), serum cholesterol (< 5.1 mmol/L, ≥ 5.1 mmol/L), and serum albumin (< 35 g/L, ≥ 35 g/L) might have an impact on retinopathy (26–30). Comorbidities such as hypertension (systolic pressure ≥130 mmHg or diastolic pressure ≥80 mmHg), liver conditions, thyroid problem, congestive heart failure (CHF), and asthma, and the use of diuretics were included because they were associated with DR or water metabolism (26, 27, 31–36). Ophthalmic imaging status (complete, partial) was also taken into account since some participants did not complete the entire imaging process due to multiple causes, including physical limitations of eyes and equipment failure.

### Statistical analysis

SAS OnDemand for Academics was applied to complete statistical analyses. Weights were utilized to match total population counts because of the complex survey design, non–response, and post-stratification adjustment. The chi-square test of the Rao-Scott modified weight was used to examine the subject characteristics at baseline, including age, sex, race, education, marital status, BMI, smoke status, alcohol consumption, blood glycohemoglobin, serum total cholesterol, serum albumin, comorbidities, use of diuretics, and ophthalmic imaging status. Multivariable logistic regression models were constructed by surveylogistic procedure with sampling weight, cluster, and strata. Model 1 was unadjusted and Model 2 was adjusted for age, sex, and race. Based on Model 2, Model 3 was further adjusted for all the other covariates. *P* value of < 0.05 or 95% confidence intervals (95% CI) that did not cross 1.00 was considered statistically significant.

## Results

### Subject characteristics

This study included 5,220 United States participants in total. Adults with DR (*n* = 641) were more likely to be older, male, of other races, with lower education level, alcohol consumption, serum total cholesterol, and serum albumin, as well as having higher BMI, glycohemoglobin, and prevalence of hypertension and CHF than adults without retinopathy (*n* = 4,579) (Table 1).

**Table 1:** Characteristics of United States participants grouped by with or without DR, NHANES 2005-2008.

**Characteristics**	**Without DR**	**DR**	***P* value**
	**(*n* = 4,579)**	**(*n* = 641)**	
**Age (years)**			< 0.001
< 65	3,014 (75.8)	372 (65.2)	
≥65	1,565 (24.2)	269 (34.8)	
**Sex**			< 0.001
Male	2,266 (46.8)	359 (55.7)	
Female	2,313 (53.2)	282 (44.3)	
**Race**			< 0.001
Non-hispanic white	2,587 (79.0)	278 (70.2)	
Other races	1,992 (21.0)	363 (29.8)	
**Education**			< 0.001
Under high school	1,247 (16.6)	247 (25.3)	
High school or equivalent	1,134 (25.8)	160 (29.4)	
Above high school	2,198 (57.6)	234 (45.3)	
**Marital status**			0.896
Married	2,960 (69.7)	414 (69.5)	
Not married	1,619 (30.3)	227 (30.5)	
**BMI (kg/m2)**			0.003
Normal weight	1,216 (28.4)	119 (20.9)	
Overweight	1,652 (35.6)	247 (36.9)	
Obese	1,711 (36.0)	275 (42.2)	
**Smoke status**			0.33
Current	924 (20.0)	121 (22.8)	
Ever	1,486 (31.3)	214 (29.2)	
Never	2,169 (48.7)	306 (48.0)	
**Alcohol consumption (g)**			0.026
0	3,146 (64.8)	479 (70.8)	
0–20	776 (18.2)	91 (16.3)	
>20	657 (17.0)	71 (12.9)	
**Glycohemoglobin (%)**			< 0.001
< 7.0	4,319 (96.3)	430 (72.8)	
≥7.0	260 (3.7)	211 (27.2)	
**Serum total cholesterol (mmol/L)**			< 0.001
< 5.1	2,125 (44.6)	351 (56.4)	
≥5.1	2,454 (55.4)	290 (43.6)	
**Serum albumin (g/L)**			0.035
< 35	56 (0.9)	19 (2.3)	
≥35	4,523 (99.1)	622 (97.7)	
**Comorbidities**			
Hypertension			< 0.001
Yes	2,189 (45.9)	384 (56.0)	
No	2,390 (54.1)	257 (44.0)	
Liver condition			0.6
Yes	205 (4.2)	25 (5.1)	
No	4,374 (95.8)	616 (94.9)	
Thyroid problem			0.992
Yes	597 (13.7)	83 (13.7)	
No	3,982 (86.3)	558 (86.3)	
History of CHF			< 0.001
Yes	169 (2.7)	63 (8.0)	
No	4,410 (97.3)	578 (92.0)	
History of asthma			0.164
Yes	546 (13.0)	99 (16.0)	
No	4,033 (87.0)	542 (84.0)	
**Use of diuretics**			< 0.001
Yes	872 (16.2)	183 (25.6)	
No	3,707 (83.8)	458 (74.4)	
**Ophthalmic imaging status**			0.225
Complete	4,429 (97.9)	613 (96.9)	
Partial	150 (2.1)	28 (3.1)	

NHANES, The National Health and Nutrition Examination Survey; BMI, body mass index.

Ns are unweighted, proportions are weighted by MEC exam weight, and P values are calculated by the chi-square test of the Rao-Scott modified weight.

### Association between serum osmolarity and DR

**Figure 2:**
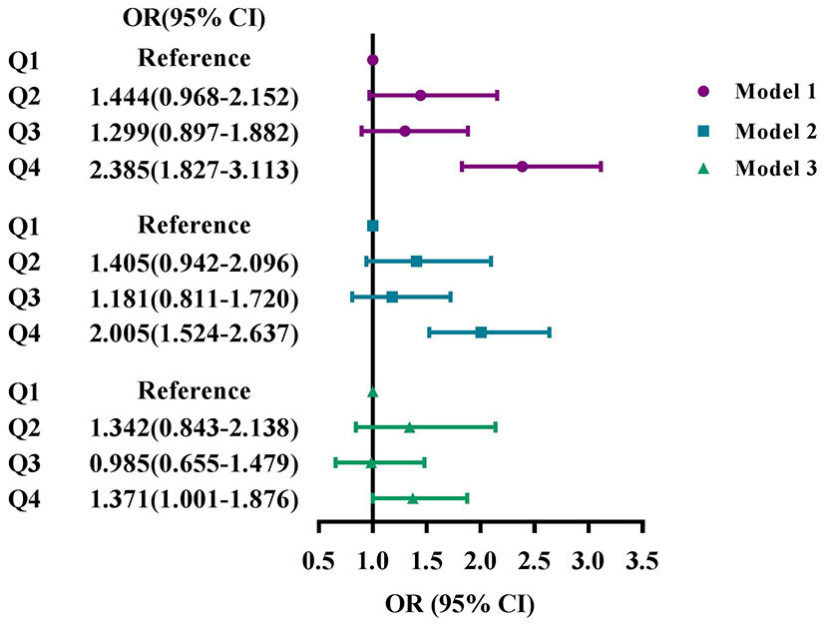
Associations of serum osmolarity with DR, NHANES 2005–2008. **(A)** Model 1: Unadjusted. **(B)** Model 2: Adjusted for age, sex, and race. **(C)** Model 3: Adjusted for age, sex, race, education, marital status, BMI, smoke status, alcohol consumption, blood glycohemoglobin, serum total cholesterol, serum albumin, comorbidities, use of diuretics, and ophthalmic imaging status. Q1, the lowest osmolality group (serum osmolality ≤ 275 mOsm/kg); Q2, the lower osmolality group (276–279 mOsm/kg); Q3, the higher osmolality group (280–282 mOsm/kg); Q4, the highest osmolality group (≥ 283 mOsm/kg).

Figure 2 showed the odds ratios (ORs) and 95% CIs for DR based on serum osmolality level. After fully adjusted, participants with the highest quartile of serum osmolarity had higher odds of DR than those in the lowest osmolality group (OR: 1.371, 95% CI: 1.001–1.876).

### Association between serum osmolarity and DR severity

Patients with DR (*n* = 641) were further classified into three groups based on the retinopathy severity, mild NPR (*n* = 521), moderate/severe NPR (*n* = 96), and PDR (*n* = 24), respectively. In the three groups, there are significant differences in race, BMI, smoke status, alcohol consumption, glycohemoglobin, serum albumin, and the use of diuretics (Table 2).

**Table 2:** Characteristics of DR patients grouped by severity, NHANES 2005-2008.

**Characteristics**	**Mild NPR**	**Moderate/severe NPR**	**PDR**	***P* value**
	**(*n* = 521)**	**(*n* = 96)**	**(*n* = 24)**	
**Age (years)**				0.333
< 65	298 (65.3)	61 (67.3)	13 (50.1)	
≥65	223 (34.7)	35 (32.7)	11 (49.9)	
**Sex**				0.456
Male	301 (56.3)	48 (50.2)	10 (59.5)	
Female	220 (43.7)	48 (49.8)	14 (40.5)	
**Race**				0.004
Non-hispanic white	247 (72.5)	26 (56.0)	5 (56.6)	
Other races	274 (27.5)	70 (44.0)	19 (43.4)	
**Education**				0.183
Under high school	191 (23.8)	43 (31.9)	13 (47.7)	
High school or equivalent	134 (30.4)	23 (26.0)	3 (9.9)	
Above high school	196 (45.8)	30 (42.1)	8 (42.4)	
**Marital status**				0.612
Married	342 (70.0)	60 (66.4)	12 (62.9)	
Not married	179 (30.0)	36 (33.6)	12 (37.1)	
**BMI (kg/m2)**				0.007
Normal weight	103 (22.6)	10 (8.3)	6 (18.5)	
Overweight	210 (38.0)	33 (31.6)	4 (22.1)	
Obese	208 (39.4)	53 (60.1)	14 (59.4)	
**Smoke status**				0.034
Current/ ever	282 (54.4)	43 (35.5)	10 (43.1)	
Never	239 (45.6)	53 (64.5)	14 (56.9)	
**Alcohol consumption (g)**				0.006
0	374 (68.2)	85 (90.0)	20 (74.1)	
0–20	82 (17.4)	6 (6.3)	3 (23.9)	
>20	65 (14.4)	5 (3.7)	1 (2.0)	
**Glycohemoglobin (%)**				< 0.001
< 7.0	394 (78.9)	30 (33.9)	6 (36.8)	
≥7.0	127 (21.1)	66 (66.1)	18 (63.2)	
**Serum total cholesterol (mmol/L)**				0.584
< 5.1	287 (55.5)	50 (60.6)	14 (68.2)	
≥5.1	234 (44.5)	46 (39.4)	10 (31.8)	
**Serum albumin (g/L)**				0.002
< 35	513 (98.7)	86 (89.6)	23 (98.6)	
≥35	8 (1.3)	10 (10.4)	1 (1.4)	
**Comorbidities**				
Hypertension				0.146
Yes	303 (54.7)	66 (63.8)	15 (65.6)	
No	218 (45.3)	30 (36.2)	9 (34.4)	
Liver condition				—
Yes	22 (5.1)	3 (5.9)	0 (0)	
No	499 (94.9)	93 (94.1)	24 (100.0)
Thyroid problem				0.399
Yes	65 (13.3)	11 (13.6)	7 (25.7)	
No	456 (86.7)	85 (86.4)	17 (74.3)	
History of CHF			0.147
Yes	44 (7.0)	12 (11.9)	7 (27.3)	
No	477 (93.0)	84 (88.1)	17 (72.7)	
History of asthma				0.877
Yes	79 (15.7)	16 (18.5)	4 (14.2)	
No	442 (84.3)	80 (81.5)	20 (85.8)	
**Use of diuretics**				0.038
Yes	136 (23.2)	37 (40.8)	10 (40.0)	
No	385 (76.8)	59 (59.2)	14 (60.0)	
**Ophthalmic imaging status**				0.287
Complete	501 (97. 2)	93 (96.4)	19 (89.3)	
Partial	20 (2.8)	3 (3.6)	5 (10.7)	

NHANES, The National Health and Nutrition Examination Survey; BMI, body mass index. Ns are unweighted, proportions are weighted by MEC exam weight, and P values are calculated by the chi-square test of the Rao-Scott modified weight.

**Figure 3:**
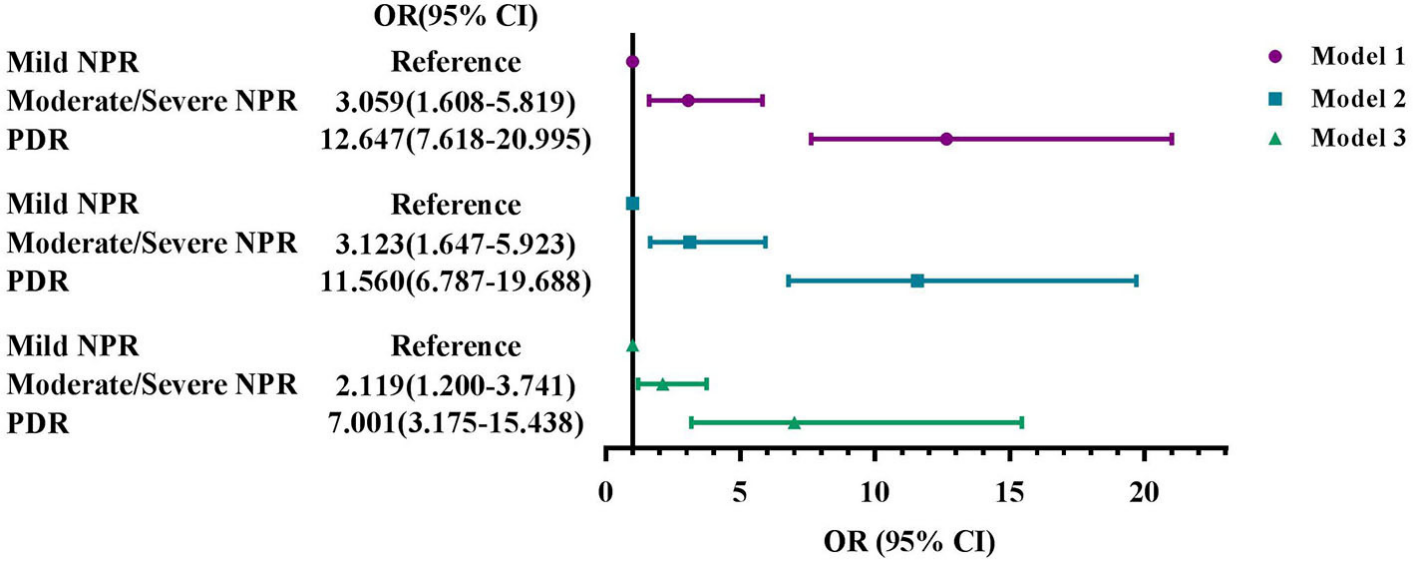
Associations of serum osmolarity with DR severity, NHANES 2005–2008. **(A)** Model 1: unadjusted. **(B)** Model 2: Adjusted for age, sex, and race. **(C)** Model 3: Adjusted for age, sex, race, education, marital status, BMI, smoke status, alcohol consumption, blood glycohemoglobin, serum total cholesterol, serum albumin, comorbidities, use of diuretics, and ophthalmic imaging status. Mild NPR, mild non-proliferative retinopathy; Moderate/severe NPR, moderate/severe non-proliferative retinopathy; PDR, proliferative retinopathy.

Figure 3 showed the association between serum osmolarity and DR severity. As the limited number of PDR patients, participants were divided into lower serum osmolarity group and higher serum osmolarity group, and liver condition was excluded from Model 3. After fully adjustment, the odds of moderate/severe NPR and PDR rose as serum osmolarity increased (OR: 2.119, 95% CI: 1.200–3.741; OR: 7.001, 95% CI: 3.175–15.438).

### Association between hydration status by calculated osmolarity and DR in older adults

**Figure 4:**
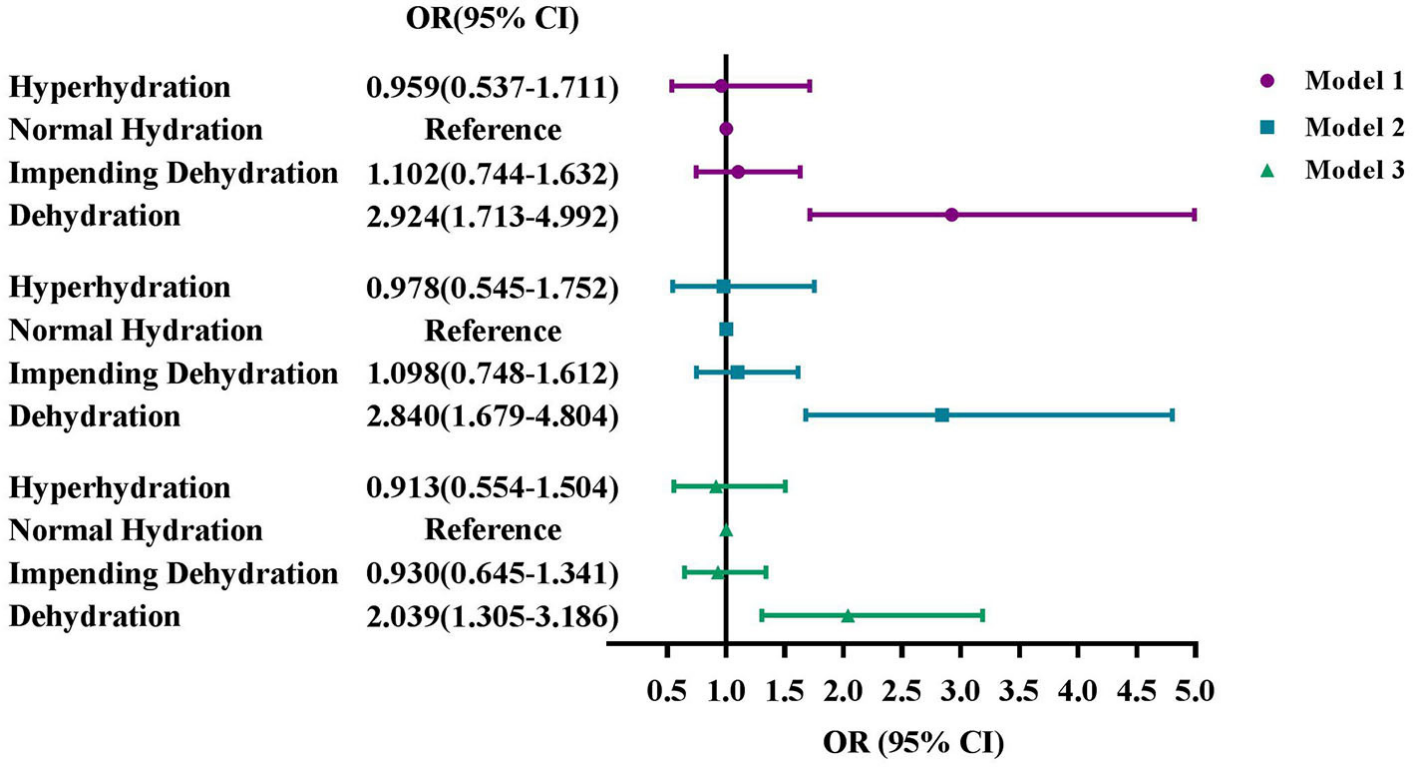
Associations of hydration status by calculated osmolarity with DR in older adults, NHANES 2005-2008. **(A)** Model 1: Unadjusted. **(B)** Model 2: Adjusted for age, sex, and race. **(C)** Model 3: Adjusted for age, sex, race, education, marital status, BMI, smoke status, alcohol consumption, blood glycohemoglobin, serum total cholesterol, serum albumin, comorbidities, use of diuretics, and ophthalmic imaging status.

In Figure 4, we sorted hydration status into four groups by calculated osmolarity. Among the 1,834 adults aged 65 years or older, 53.3% of them (*n* = 978) had normal hydration, 29.7% adults were of impending dehydration (*n* = 545), 11.0% adults were dehydrated (*n* = 201), and 6.0% adults were of hyperhydration (*n* = 110). Similar trends could be seen in Model 1, 2, and 3. After fully adjusting, dehydration was significantly associated with DR (OR: 2.039, 95% CI: 1.305–3.186), whereas impending dehydration or hyperhydration had no significant differences with DR.

## Discussion

From this cross-sectional study based on NHANES 2005–2008, we found the association between hydration status and DR. Participants with higher serum osmolarity were more likely to develop DR and to have more serious DR levels.

When we used serum osmolality, the gold standard currently, to evaluate hydration status in adults, only 0.1% of them were dehydrated. However, 6.8% participants had dehydration by using calculated osmolarity. The explanations for these differences are as follows. Firstly, comorbidities including hyperglycemia, hypertension, and hyperlipidemia might have a greater impact on DR than dehydration. Blood glucose and urea nitrogen levels were more emphasized in calculated osmolarity. It seemed that in diabetic kidney disease patients with albuminuria, glomerular filtration rate and albumin excretion rate were independently associated with DR (37). Secondly, serum osmolality above 300 mOsm/kg was the diagnostic criteria of hypotonic dehydration (15). Salt and water balance should also be taken into account when serum osmolality was at a normal level, as isotonic or hypotonic dehydration might also be existed (38, 39). For patients with mild to moderate dehydration, oral or enteral rehydration and subcutaneous infusion are the main therapy methods (9). Parenteral administration of fluids and electrolytes is the preferred treatment for severe dehydration (40). However, dehydration could be prevented by consuming adequate fluid and keeping the balance of fluids and electrolytes (41, 42). Adequate fluid intake could be achieved by providing straws and cups, offering fluids regularly, using frozen juice bars, and increasing awareness of factors responsible for dehydration such as fever, hot weather, vomiting and diarrhea (9).

The results of this study can be explained by the following mechanisms. Water makes up a quite high proportion of our eyes, with the highest concentrations in aqueous and vitreous humors (about 98–99% by volume) (10, 43). The occurrence and progression of DR is related to complex vascular, inflammatory, and neuronal mechanisms (44). Hydration status played an important role in the pathophysiological process of DR. Madonna, et al. demonstrated that glucose-related hyperosmolarity could promote retinopathy in type 1 diabetic mice (45). Blood-retinal barrier (BRB) was composed of retinal endothelial cells and retinal pigment epithelial (RPE) cells, which played an important role in water and ion balance (46, 47). With the increase of serum osmolarity, the RPE may be affected by hyperosmolar stimulus, resulting in a breakdown of BRB (47, 48). Simultaneously, high serum osmolality influences the function of RPE in eliminating water from subretinal space, resulting in retinal edema (47). In addition, rapid water transport by Müller glial cells may result from dehydration of the outer retina, and this alteration may lead to edema in the post-ischaemic retina (49). Retinal edema is a risk factor of retinal injury especially when the retina is in an inflammatory and ischaemic state (49, 50).

In a real-life pilot study, dehydration caused by prolonged physical effort may alter the retinal structure and its vascularization, leading to a low ocular blood flow rate (51). NPR is caused as a result of altered retinal blood flow and vascular permeability, thickened basement membrane, lost pericytes, and formed acellular capillary (26, 52–54). Dehydration also contributed to the up-regulation of interleukin-6 (IL-6) in the supraoptic and paraventricular regions, which lead to an increase in IL-6 secretion (55–57). IL-6, a potent angiogenic factor, is significantly associated with DR severity, especially in patients with PDR (58–60).

This study has several strengths. To the best of our knowledge, this is the first study to investigate the association between hydration status and DR. We used NHANES data, which contained the nationally representative samples of adult population in United States. We also used serum osmolality, the gold standard, to assess hydration status in order to ensure the accuracy of the results. Moreover, elderly people frequently dehydrate, especially those who reside in care facilities (23, 61). The results of this study remind us of paying closer attention to hydration status, which may help to delay the progress of DR.

The limitations of this study should also be addressed. Firstly, this is a cross-sectional study. Despite there is a significant association between hydration status and DR, the cause-effect relationship is still unclear. Secondly, the proportion of dehydration was low, therefore we divided the data into quartiles to identify different levels of hydration status. As a result, we lacked a judgment criterion for abnormal hydration status, making it challenging to estimate the risk of DR from dehydration in real life. Thirdly, we only considered the most common form—hypertonic dehydration, which could be measured by serum osmolality directly. Isotonic or hypotonic dehydration is hard to diagnose by the currently available data. Moreover, although non-mydriatic digital retinal images are not the gold standard for DR diagnosis, they can be equally used to screen the diabetic population and grade DR severity (62). Finally, because the retinal imaging exam was only performed from 2005 to 2008, the survey data might not be suitable for current conditions.

## Conclusions

In conclusion, this study revealed a significant positive association between hydration status and DR. The severity of DR increased as the serum osmolality rose. For people at risk for DR, it is critical to pay close attention to their hydration status in order to prevent and delay the occurrence of vision loss. For diabetics and older adults, adequate fluid intake is essential. Meanwhile, physicians are urged to correct their patients' abnormal hydration status timely. Furthermore, teleophthalmology, an accurate, fast, and affordable method for DR screening, has developed rapidly and may help reduce the burden of deteriorating DR due to the difficulty of monitoring and timely treatment in specialized medical institutions in the future (63, 64).

## Data availability statement

The original contributions presented in the study are included in the article/supplementary material, further inquiries can be directed to the corresponding authors.

## Ethics statement

The studies involving human participants were reviewed and approved by the National Center for Health Statistics Research Ethics Review Board. The patients/participants provided their written informed consent to participate in this study.

## Author contributions

JZ: conceptualization, data analysis, and writing-original draft. ZR and JL: data curation and validation. JZ, ZR, QZ, RZ, CZ, and JL: revision, editing the manuscript, read, and approved the final manuscript.

## Conflict of interest

The authors declare that the research was conducted in the absence of any commercial or financial relationships that could be construed as a potential conflict of interest.

## Publisher's note

All claims expressed in this article are solely those of the authors and do not necessarily represent those of their affiliated organizations, or those of the publisher, the editors and the reviewers. Any product that may be evaluated in this article, or claim that may be made by its manufacturer, is not guaranteed or endorsed by the publisher.
